# Demographic Data Associated With Digital Inequity Reported in Patient-to-Provider Teledermatology Studies in the United States From 2011 to 2021: Scoping Review

**DOI:** 10.2196/43983

**Published:** 2023-02-28

**Authors:** John Miller, Patrick Ioffreda, Shannon Nugent, Elizabeth Jones

**Affiliations:** 1 Sidney Kimmel Medical College, Thomas Jefferson University Philadelphia, PA United States; 2 Department of Dermatology and Cutaneous Biology Sidney Kimmel Medical College Thomas Jefferson University Philadelphia, PA United States

**Keywords:** teledermatology, equity, inequity, scoping review, digital divide, dermatology, racial minority, ethnic minority, digital inequity, patient care, COVID-19

## Abstract

**Background:**

Patient-to-provider teledermatology relies on a patient’s access to technology to ensure a successful visit. However, access to broadband internet and technology varies across populations in the United States—leading to the digital divide. While teledermatology has been recognized as a model to improve access, little is known about how often demographic data associated with digital inequity are captured in studies.

**Objective:**

Given the expansion of teledermatology over the past decade, we sought to determine how often demographic data associated with digital inequity are reported in patient-to-provider teledermatology studies.

**Methods:**

A scoping literature review search was conducted using the search term *teledermatology* in the following databases: PubMed, Embase, and the Cochrane Database of Systematic Reviews. All studies published between December 31, 2011, and December 31, 2021, that evaluated patient-to-provider teledermatology were eligible.

**Results:**

In total, 1412 publications describing teledermatology were identified, of which 46 met the inclusion criteria. Race or ethnicity was the most frequently reported demographic characteristic (28/46, 61%). However, only 41% (19/46) of studies were representative of race or ethnicity, defined as including >20% nonwhite participants. Studies rarely reported the number of participants greater than 65 years of age (14/46, 30%), preferred language (9/46, 20%), income (6/46, 13%), highest level of education (5/46, 11%), or access to a device (4/46, 9%). Studies conducted after the onset of the COVID-19 pandemic were significantly more likely to report preferred language (9/25, 36% vs 0%; *P*=.002) and appeared more likely to report other demographic data associated with digital inequity, without reaching statistical significance (*P*>.05).

**Conclusions:**

Demographic data associated with digital inequity are rarely reported in patient-to-provider teledermatology studies to date. These studies frequently lack adequate representation of racial and ethnic minorities. With increased calls for equitable representation in dermatology studies, future teledermatology studies can improve the reporting of race and ethnicity and consider demographic data associated with digital inequity as an important criterion in research design.

## Introduction

Unlike traditional in-person care, patient-to-provider teledermatology relies on a patient’s access to a compatible device and broadband internet to ensure a successful visit. However, access to broadband internet and technology varies across populations in the United States—creating what is referred to as the digital divide [1,2]. Populations less likely to report a broadband internet subscription include those with less formal education or lower income, racial and ethnic minorities, persons greater than 65 years of age, and residents of rural communities [3].

Demographics that report less access to broadband internet and technology recorded lower telehealth utilization rates during the COVID-19 pandemic. For instance, older age and persons who reported a non-English language as their primary language were associated with fewer completed telemedicine visits [4-6]. Inequity in access to telemedicine care exists. However, it is unknown whether demographic data associated with digital inequity are frequently reported in teledermatology studies.

Demographic data associated with digital inequity should be included in patient-to-provider teledermatology studies to ensure conclusions that reflect all populations. We conducted a scoping review of patient-to-provider teledermatology studies conducted in the United States to characterize how often patients vulnerable to the digital divide are represented. Given the expansion of teledermatology during the COVID-19 pandemic, we also compared how often demographic data associated with digital inequity were reported in studies conducted prior to and after the declaration of the pandemic.

## Methods

### Study Design

A scoping review search was performed on PubMed, Embase, and the Cochrane Database of Systematic Reviews from December 31, 2011, through December 31, 2021. We used the search term *teledermatology*. This study is registered with the International Prospective Register of Systematic Reviews (CRD42022325030) and followed the relevant portions of the PRISMA-ScR (Preferred Reporting Items for Systematic Reviews and Meta-Analyses extension for Scoping Reviews) checklist.

### Eligibility Criteria

Studies were included if they evaluated an asynchronous or synchronous model of patient-to-provider teledermatology. Both quantitative and qualitative studies evaluating teledermatology were included to represent the broad scope of research. Only studies conducted in the United States were included. No restrictions were placed on the age or gender of study participants. Telephone-based studies were excluded as these encounters involve separate technology and resources unrelated to measures of broadband connectivity and access to a device.

### Article Selection and Data Extraction

Two reviewers (PI and JM) sequentially evaluated titles, abstracts, and then the full text of all publications based on the inclusion criteria. Studies were assessed based on whether they reported demographic data associated with digital inequity as described in a national report by the Pew Research Center [3]. These included participant income, highest level of education, race or ethnicity, number of participants greater than 65 years of age, and residents of rural communities [3]. Studies were defined as unrepresentative of race and ethnicity if they included less than 20% nonwhite participants, consistent with the methodology of a previous study by Chen et al [7].

### Data Analysis and Synthesis

The primary outcome was the frequency of reported demographic characteristics associated with digital inequity. The secondary outcome was the likelihood of reporting demographic characteristics associated with digital inequity in studies conducted prior to versus during the COVID-19 pandemic. The Fisher exact test was used to analyze the secondary outcome. Analysis was performed using Stata software (version 17.0; StataCorp LLC). *P*<.05 was considered statistically significant.

## Results

### Selection of Articles

**Figure 1:**
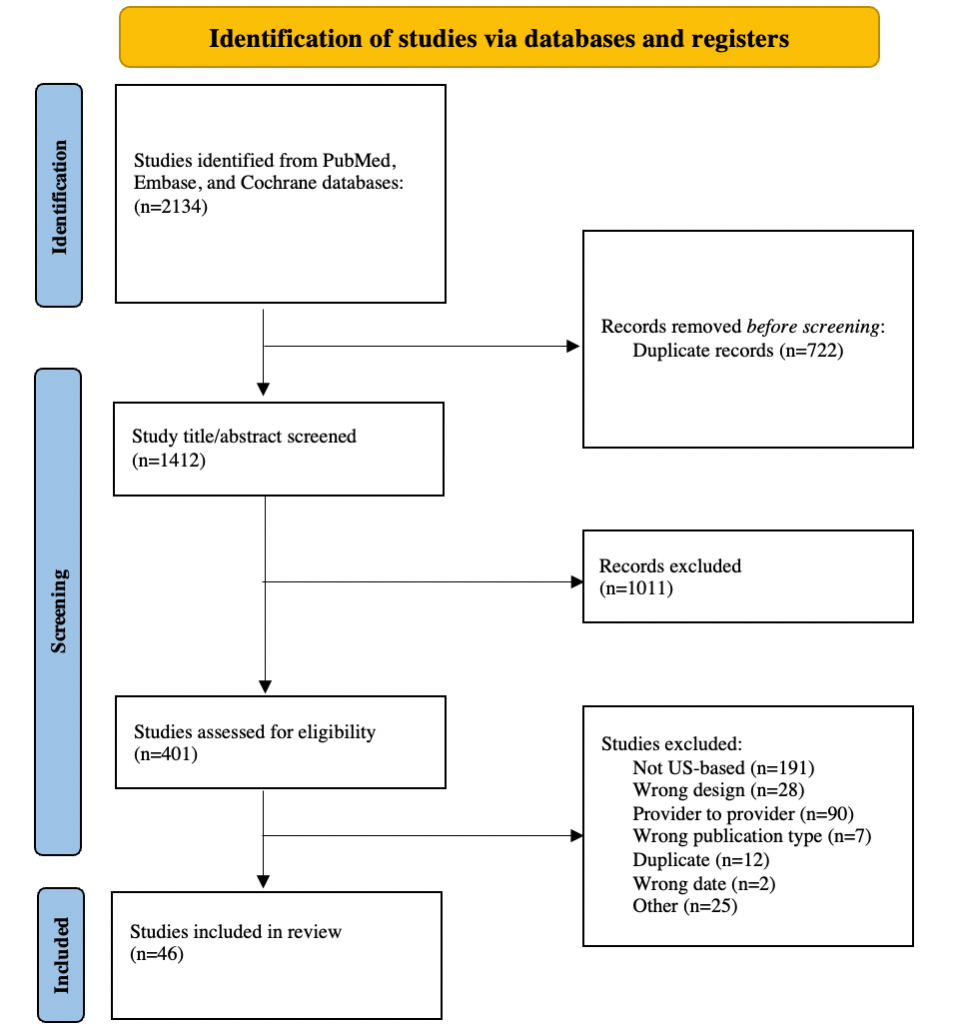
The PRISMA (Preferred Reporting Items for Systematic Reviews and Meta-Analyses) flowchart for publication selection.

Among 1412 potential studies, 46 satisfied the inclusion criteria [8-53]. Figure 1 shows the PRISMA (Preferred Reporting Items for Systematic Reviews and Meta-Analyses) flowchart illustrating the publication selection process. The full list of included studies and general study characteristics are presented in Multimedia Appendix 1.

### Demographic Data Associated With Digital Inequity

Demographic data associated with digital inequity reported in patient-to-provider teledermatology studies are presented in Table 1. Studies most often reported race or ethnicity (28/46, 61%), but a representative sample was present in only 41% (19/46). Of 46 studies, 14 (30%) reported the number of participants greater than 65 years of age. Studies infrequently reported preferred language, income, highest level of education, or access to a device (Table 1). No studies reported participant-reported internet quality or the number of rural residents included.

**Table 1:** Demographic data reported in patient-to-provider teledermatology studies.

Characteristic reported	Studies (N=46), n (%)
Race or ethnicity	28 (61)
Representative of racial and ethnic minorities^a^	19 (41)
Number of participants >65 years of age	14 (30)
Preferred language	9 (20)
Income	6 (13)
Highest level of education	5 (11)
Access to a device	4 (9)
Internet quality	0 (0)
Number of rural residents	0 (0)

^a^Studies were representative of racial and ethnic minorities if they included >20% nonwhite participants.

### Demographic Data Reported and COVID-19

Demographic data reported in patient-to-provider teledermatology studies conducted before (n=21) and after (n=25) the declaration of the COVID-19 pandemic are presented in Table 2. Studies conducted after the onset of the COVID-19 pandemic appeared more likely to report race or ethnicity, number of participants older than 65 years, income, highest level of education, and access to a device, without reaching statistical significance. Studies conducted during the COVID-19 pandemic were significantly more likely to report the participant’s preferred language (9/25, 36% vs 0%; *P*=.002). Studies conducted before and after the declaration of the COVID-19 pandemic were representative of racial and ethnic minorities at comparable rates.

**Table 2:** Demographic data reported in patient-to-provider teledermatology studies conducted before and after the declaration of the COVID-19 pandemic.

Characteristic reported	Studies conducted before COVID-19 (n=21), n (%)	Studies conducted after COVID-19 (n=25), n (%)	*P* value
Race or ethnicity	11 (52)	17 (68)	.22
Representative of racial and ethnic minorities^a^	9 (43)	10 (40)	.54
Number of participants >65 years of age	4 (19)	10 (40)	.11
Preferred language	0 (0)	9 (36)	.002
Income	2 (10)	4 (16)	.42
Highest level of education	1 (5)	4 (16)	.23
Access to a device	1 (5)	3 (12)	.37

^a^Studies were representative of racial and ethnic minorities if they included >20% nonwhite participants.

## Discussion

### Principal Findings

The findings of this study highlight that demographic data associated with digital inequity are infrequently included in patient-to-provider teledermatology studies. With the explosion of telemedicine during the pandemic, our study noted a promising trend toward the inclusion of patient demographic information associated with digital inequity regarding race or ethnicity, participants older than 65 years, income, highest level of education, and access to a device. Preferred language was the only demographic characteristic that was significantly more likely to be reported in teledermatology studies following the pandemic onset. Even so, an overall lack of reporting of demographic data associated with digital inequity persisted.

### Demographic Data Associated With Digital Inequity

Demographic data associated with digital inequity may be an indicator of the likelihood to complete teledermatology visits. Patients greater than 65 years of age, those who reported a non-English language as their primary language, and participants with lower income were less likely to complete teledermatology visits compared to their counterparts [5,6,8,9]. Other studies noted patients older than 65 years and those reporting lower income were less satisfied with the visit experience [10-12]. Demographic data associated with digital inequity should be reported more often to inform inclusive research design and ensure generalizable conclusions.

The deficiencies in racial and ethnic representation identified in this review are consistent with those reported in dermatology clinical trials over the past decade [7]. It is critical to capture race and ethnicity data in patient-to-provider teledermatology studies as Black race and Latinx ethnicity were associated with lower use of video-based telemedicine during the pandemic [4]. Future teledermatology studies can improve the reporting of race and ethnicity and should consider this important criterion in research design.

### Demographic Data Reported and COVID-19

Our study noted a trend toward the inclusion of demographic data associated with digital inequity in studies conducted after the COVID-19 pandemic compared to before. Dermatologists quickly adopted and expanded their teledermatology programs at the onset of the pandemic [54]. The sheer increase in the volume of care due to a lack of access to alternatives and improved reimbursement likely drove a wider demographic reach in the patient population. This trend coincides with increased calls for equitable inclusion in dermatology studies [55] and studies highlighting how teledermatology may exacerbate digital inequity [4,56-60]. As a result, lack of access combined with increased awareness of social inequities in care possibly encouraged researchers to be more intentional in seeking broader demographic variables to ensure their teledermatology programs represented underserved and underrepresented communities. Despite the positive trend, reporting of inclusive demographic data is insufficient to date and can be improved upon in future research design.

### Limitations

This study is limited in its design. The studies included in our review had variable primary outcomes. Of note, our study did not determine whether a study’s primary outcome correlated with the inclusion of demographic data associated with digital inequity. It is also possible that studies captured demographic data associated with digital inequity in their methods but did not report these findings. This trend is consistent with medical studies’ failure to report sociodemographic variables consistently [61]. Studies will be unable to correct health inequity if these variables are not reported more consistently [61]. Lastly, this study only reflects demographic reporting trends in studies conducted in the United States.

### Conclusion

This study highlights the need for equitable representation of racial and ethnic minorities and improved reporting of demographic data associated with digital inequity in patient-to-provider teledermatology studies over the past decade. With increased calls for equitable representation in dermatology studies, future teledermatology studies can improve reporting of race and ethnicity and consider the inclusion of demographic data associated with digital inequity an important criterion in research design. Professional and telehealth societies can help drive change by creating guidelines that reflect the value of reporting demographic data associated with digital inequity. Continued research and education can increase awareness of the digital divide’s impact on patient care and help optimize teledermatology’s potential to serve vulnerable populations.

## Appendix

Multimedia Appendix 1 General characteristics of included studies.

## Abbreviations

PRISMA: Preferred Reporting Items for Systematic Reviews and Meta-Analyses
PRISMA-ScR: Preferred Reporting Items for Systematic Reviews and Meta-Analyses extension for Scoping Reviews
