# Supplementary material for: Demographic Data Associated With Digital Inequity Reported in Patient-to-Provider Teledermatology Studies in the United States From 2011 to 2021: Scoping Review
Source: JMIR Dermatol. 2023 Feb 28;6:e43983. doi: 10.2196/43983 (PMC10012205; doi:10.2196/43983)
Supplement: Multimedia Appendix 1 [file derma_v6i1e43983_app1.docx]

Table 1. General characteristics of included studies

| **Author** | **Teledermatology Model Studied** | **Design** | **Primary Outcome** | **Number of participants** |
| --- | --- | --- | --- | --- |
| Miller [8] | Synchronous | Retrospective | Descriptive | 250 |
| Blundell [9] | Asynchronous | Retrospective | Descriptive | 1078 |
| Yeroushalmi [10] | Synchronous | Retrospective | Satisfaction | 168 |
| Su [11] | Synchronous | Retrospective | Descriptive | 3,982 |
| Kaunitz [12] | Synchronous | Retrospective | Satisfaction | 602 |
| Hamad [13] | Synchronous | Retrospective | Satisfaction | 184 |
| Pearlman [14] | Synchronous | Retrospective | Satisfaction | 100 |
| Afanasiev [15] | Hybrid | Retrospective | Descriptive | 74,411 |
| Cline [16] | Hybrid | Retrospective | Descriptive | 305 |
| Vasavda [17] | Synchronous | Retrospective | Descriptive | 14,334 |
| Gu [18] | Synchronous | Retrospective | Descriptive | 505 |
| Cline [19] | Synchronous | Retrospective | Descriptive | 228 |
| Franciosi [20] | Synchronous | Retrospective | No Show Visit Rate | 6,883 |
| Haevale [21] | Synchronous | Retrospective | Descriptive | 1,199 |
| Krueger [22] | Asynchronous | Retrospective | Descriptive | 2,292 |
| Pannu [23] | Synchronous | Prospective | Satisfaction | 305 |
| Moore [24] | Synchronous | Retrospective | Satisfaction | 171 |
| Das [25] | Hybrid | Retrospective | Isotretinoin prescription dose | 143 |
| Drugge [26] | Asynchronous | Retrospective | Descriptive | 1,675 |
| Johnson [27] | Asynchronous | Prospective | Identification of barriers to teledermatology access | 33 |
| Hekman [28] | Synchronous | Retrospective | Satisfaction | 46 |
| Asabor [29] | Synchronous | Retrospective | Satisfaction | 548 |
| Kazi [30] | Synchronous | Retrospective | Descriptive | 2,623 |
| McGee [31] | Synchronous | Retrospective | Descriptive | 274 |
| Yi [32] | Synchronous | Retrospective | Descriptive | 1,415 |
| Edison [33] | Synchronous | Prospective | Provider communication | 92 |
| Creighton-Smith [34] | Asynchronous | Retrospective | Diagnostic Concordance | 1021 |
| Parsi [35] | Asynchronous | Prospective | Cost-effectiveness | 64 |
| Fathy [36] | Synchronous | Retrospective | Antibiotic prescription patterns | 9,007 |
| Chambers [37] | Asynchronous | Prospective | Treatment concordance | 64 |
| O’Connor [38] | Asynchronous | Prospective | Diagnostic Concordance | 40 |
| Kornmehl [39] | Asynchronous | Prospective | Quality of Life | 156 |
| Armstrong [40] | Asynchronous | Prospective | Treatment concordance | 156 |
| Armstrong [41] | Asynchronous | Prospective | Treatment Concordance | 296 |
| Armstrong [42] | Asynchronous | Prospective | Quality of Life | 296 |
| Ford [43] | Asynchronous | Prospective | Access | 148 |
| Fiks [44] | Asynchronous | Prospective | Utilization | 197 |
| Khosravi [45] | Asynchronous | Retrospective | Follow-up rate | 400 |
| Clark [46] | Synchronous | Retrospective | Descriptive | 779 |
| Gregory [47] | Asynchronous | Retrospective | Descriptive | 452 |
| Lamel [48] | Synchronous | Retrospective | Descriptive | 1500 |
| Bosanac [49] | Asynchronous | Prospective | Satisfaction | 26 |
| Rajda [50] | Asynchronous | Prospective | Descriptive | 243 |
| Wu [51] | Asynchronous | Prospective | Diagnostic concordance | 34 |
| Jacoby [52] | Asynchronous | Prospective | Diagnostic concordance | 14 |
| Pathipati [53] | Asynchronous | Prospective | Satisfaction | 38 |

^a^ Descriptive studies included those that analyzed trends in teledermatology utilization rates among different demographics and ICD codes associated with visits
